# 

**Published:** 1860-06

**Authors:** 


### CONTENTS FOR JUNE, 1860

### ORIGINAL COMMUNICATIONS.

The Mutual Relations and consequent Mutual Duties of the Medical Profession and the Community. A Popular Lecture before the Illinois State Medical Society. By N. S. DAVIS, M. D. .......... 321
Pathology of Neuralgia. By L. D. ROBINSON, M. D., of New Elizabeth, Ind. .......... 333
Reports of Surgical Cases. By E. ANDREWS, M. D., of Chicago, .......... 338
Report of the Proceedings of the Tenth Annual Meeting of the Illinois State Medical Society .......... 342

### BOOK AND PAMPHLET NOTICES.

The Life of John Collins Warren, M. D., compiled chiefly from his Autobiography and Journals. By EDWARD WARREN, M. D., .......... 356
The Medical Uses of Electricity in the Treatment of Nervous Affections .......... 357
Transactions of the Thirty-Sixth Annual Meeting of the Medical Society of Virginia, .......... 357
Historical and Biographical Address, Delivered before the Cortland County Medical Society, N. Y., at the Fiftieth Anniversary Meeting, Aug. 10th, 1858 .......... 358
Diphtheritis; A concise Historical and Critical Essay on the late Epidemic Pseudo Membraneous Sore-Throat, of California, (1856-7) with a few remarks illustrating the Diagnosis, Pathology and Treatment of the Disease. By V. J. FOURGEAND. M. D, .......... 358
On the difficulties and advantages of Catheterism of the Air-Passages in Diseases of the Chest. By HORRACE GREEN, M. D., L. L. D., etc .......... 358

### SELECTIONS.

Biography of Claude Bernard, .......... 359
Virchow's Views on Cellular Pathology, .......... 364
The Uterus and its Inflexions, .......... 367
On the Origin of Uterine Flexions, .......... 368
On the difficulty of the Diagnosis between Pregnancy and Tumors of the Abdomen, .......... 369
Practical Remarks on Foetal Auscultation, .......... 371
The Mental Peculiarities and Disorders of Childhood, .......... 372
On the Employment of Iodide of Potassium in Diseases of the Brain in Children, .......... 374
Quinine,—Its Remedial Powers, .......... 375

### EDITORIAL.

On Consumption, .......... 380
Tenth Annual Meeting of the Illinois State Medical Society, .......... 381
Apology, .......... 383
Another New Medical School, etc .......... 383

